# Antiphospholipid Antibodies and Antiphospholipid Syndrome during Pregnancy: Diagnostic Concepts

**DOI:** 10.3389/fimmu.2015.00205

**Published:** 2015-05-07

**Authors:** Roger A. Levy, Flavia Cunha dos Santos, Guilherme R. de Jesús, Nilson R. de Jesús

**Affiliations:** ^1^Department of Rheumatology, Universidade do Estado do Rio de Janeiro, Rio de Janeiro, Brazil; ^2^Department of Obstetrics, Universidade do Estado do Rio de Janeiro, Rio de Janeiro, Brazil

**Keywords:** antiphospholipid syndrome, antiphospholipid antibodies, recurrent early miscarriage, fetal death

## Abstract

Antiphospholipid syndrome (APS) comprises of a wide spectrum of clinical and obstetric manifestations linked to the presence of antiphospholipid antibodies (aPL). APS was described in the context of lupus, and later as an isolated syndrome or primary APS. The presence of aPL, especially the lupus anticoagulant test, is associated with adverse pregnancy outcomes, such as fetal death, recurrent early miscarriages, pre-eclampsia, and placental insufficiency, but does not seem to influence infertility. High quality scientific data to support these associations, however, are lacking, and controversies arise about the definition of positive aPL (low vs medium-high titers) or even the definition of the adverse events. This review discusses APS classification criteria and the current debate about it.

## Background

Antiphospholipid syndrome (APS) involves clinical (venous and arterial thrombosis) and obstetric manifestations related to the presence of antiphospholipid antibodies (aPL). APS was described in patients with systemic lupus erythematosus (SLE), and later as an isolated syndrome or primary APS (PAPS). The classification criteria were designed for the definition of APS to be used by epidemiologic and clinical studies (1), but are commonly misused for clinical diagnostic decisions on an individual basis. According to the APS criteria, obstetrical findings are: one or more unexplained deaths of a morphologically normal fetus at or beyond the 10th week of gestation, with normal fetal morphology documented by ultrasound or by direct examination of the fetus; or one or more premature births of a morphologically normal neonate before the 34th gestational week because of eclampsia or severe pre-eclampsia according to standard definitions, or recognized features of placental insufficiency (PI); or three or more unexplained consecutive spontaneous abortions before the 10th gestational week; maternal anatomic, hormonal abnormalities, and parental chromosomal causes excluded (1).

The aPL tests included in the revised criteria for APS classification are: anti-cardiolipin (aCL) and anti-β2 glycoprotein I (aβ2GPI) antibodies, and the lupus anticoagulant (LA); their presence should be confirmed at least 12 weeks apart. (1). Other autoantibodies directed to proteins involved in the coagulation cascade or their complex with other phospholipids, or to β2GPI specific domains, have been proposed to be relevant but their clinical utility and diagnostic value remain unclear. The clinical relevance of IgA aCL or β2GPI, and if it should be included in the routine diagnostic algorithm is being discussed (2).

The mechanisms of these complications are linked to events involving several phases of the clotting system, and affecting different vessel sizes and organs. In addition to classical thrombotic mechanism, causing fetal death due to decidual vessel thrombosis, aPL have been associated to complement activation, reduction of annexin-V, and placental tissue damage, ultimately resulting in abortion (3). These findings are correlated to the pathogenic mechanism of recurrent early miscarriage (REM), pre-eclampsia, and PI (4). Other obstetrical complications that can occur during aPL/APS pregnancy such as fetal growth restriction and fetal distress do not have a clear thrombotic cause (4).

Recent research suggests that not all aPL confer the same degree of risk; these findings that may help guide future research and treatment. Among the aPL, LA is the most powerful predictor of pregnancy loss. However, aCL and aβ2GPI are also associated with adverse outcome. Of the aCL isotypes, IgG carries more risk than IgM, and IgA is not an independent predictor. In addition, other clinical features, specifically concomitant SLE, contribute to pregnancy risk. Predictors of events in APS patients or aPL carriers are prior thrombosis history, smoking, and triggering factors such as surgery, trauma, and severe infections of any kind.

### Fetal death

The relationship between high IgG aCL titers and the chance of fetal loss is well established, especially if there is a past history of fetal deaths. A multicenter prospective observational study reported that LA was the primary predictor of adverse pregnancy outcome after 12 weeks of gestation, including fetal death (5). The association of fetal death and aPL was the focus of a systematic literature review of 2011 (6), and each antibody (LA, aCL, and aβ2GPI) had positive association. Nevertheless, the authors highlight several limitations of the studies.

First, the definition of fetal death was heterogeneous, and several studies included patients with abortion and stillbirth in the same group of analysis, although an association was found with both stillbirth (>20 weeks of gestation) and fetal death (>10 weeks of gestation). This result suggests that aPL can result in pregnancy loss during different stages of pregnancy. The other limitations found were a wide methodological discrepancy in aPL assays, in the cut-off values applied to the different assays, and frequently IgG and IgM values were combined. Finally, only a small number of studies strictly followed the recommendations of the consensus regarding laboratory tests (1).

The Stillbirth Collaborative Research Network (SCRN) conducted a large, multicenter, multiethnic prospective population-based study in the US to improve current knowledge considering the association between aPL and stillbirth (7). Elevated aCL and aβ2GPI levels were associated with 3- to 5-fold increased odds of stillbirth in 582 cases of fetal death beyond the 20th week of gestation. LA, however, was not tested, and longitudinal confirmation of the results was not performed to conclude the diagnosis of APS. Advantages of this study compared to previous ones are the inclusion of a significant number of cases and the laboratory centralization for homogeneous testing.

Subsequent qualitative analysis of published studies was performed by the 14th International Congress on Antiphospholipid Antibodies Obstetric Task Force Group (APLA-ObTF), investigating patients with complete aPL profile. In their literature review, 10 published studies that performed all the three criteria tests in women with fetal death were retrieved, and the results suggest that aPL profile may be important to obstetric APS. Triple aPL positivity was identified as a risk factor for pregnancy failure by several studies, and patients with this aPL profile are more likely to have an unequivocal laboratory diagnosis and higher chance to present the most pathogenic antibodies (8).

There is an ongoing debate about aCL and aβ2GPI titers in patients with fetal loss, as well with REM. The results of published studies considering low titers of aPL in pregnancy are conflicting, ranging from good outcome in almost 80% of cases (9) to poor obstetric results just like patients with medium-high titers (10). Other studies, including the SCRN, also described a possible association between low titer aPL and obstetric events, but a causal relationship was not confirmed. A different point of view is that pathologically irrelevant low titer aPL can be found in woman with fetal death.

To alleviate part of these uncertainties, the APLA-ObTF proposed that the most reasonable approach to collect data about aPL-associated fetal death is through the use of detailed multinational registries of prospectively followed pregnancies, using centralized certified laboratories (8). Also, association with aPL profile (triple vs double vs single positivity) and aPL titer (low vs high titers) should be investigated in future studies.

### Recurrent early miscarriages

According to the APS classification criteria (1), three or more REM are classified, but many physicians investigate aPL in women with two early miscarriages. Initially, other identifiable triggers of REM, which can occur in up to 3% of all fertile women, must be ruled out. Chromosomal abnormalities in either partner can account for 4% of the causes, while abnormal embryonic karyotype in REM has been reported as frequent as in sporadic abortions (11). The real prevalence – and association – of uterine malformations, infections, other autoimmune disorders (thyroiditis, celiac disease), and congenital thrombophilias with REM is not precisely known. APS or aPL can be linked to approximately 15% of the REM, meaning that many times there is no identifiable cause.

Experimental studies support the association of aPL and REM, with different proposed mechanisms (3). Several prevalence studies in the last decades reported a high frequency of aPL in patients with REM compared to healthy controls (8), but significant methodological limitations can be found in these papers. Here again, the importance of using the standardized tests and follow the rule of considering the cut-off for samples in the moderate to high range as true positives must be emphasized. The definition of aPL positivity among studies was unequal and most did not follow international criteria recommendations (1). As with fetal loss, controversial results have been yielded between low titers of aPL and REM, and a definite association is not possible. Also, confirmation of persistent positivity and tests for all three antibodies were seldom performed, with LA and aCL being more frequently investigated.

Almost 30% of the studies included in the mentioned literature review (8) regarding the association of aPL with REM included patients with less than three abortions, and a significant number of the publications did not state if the events were consecutive. As mentioned before, the inclusion of patients with early losses (<10 weeks) and late abortion or stillbirths in the same group of analysis could be a confounding factor, since the pathogenesis of pregnancy loss varies throughout pregnancy. Furthermore, exclusion of other causes of abortion was not clearly stated in these studies.

With all these limitations, a meta-analysis could only include two studies (907 patients) to appraise the association between REM and aPL (12). They found a positive association of aCL IgG (low and moderate to high titers) and REM before 13 weeks’ gestational age, but LA and aCL IgM relationship could not be evaluated due to lack of studies that followed meta-analysis’ inclusion criteria. The literature review presented during by the APLA ObTF reported that most studies (27/46) found a positive association between REM and aPL, but only four papers strictly followed REM criteria as described in international consensus.

Most publications considering aPL link to REM reported a positive association, but with a huge heterogeneity that hinders comparisons. The APLA-ObTF members concluded that multicenter studies using current international criteria, including standardized antibodies tests, should be developed to improve our knowledge about the relationship between aPL and REM.

Well-designed studies should be performed to further understand the association between aPL and two consecutive early abortions, or low titers of aPL with REM. That said, we do not agree with some recommendations to diagnose patients that do not fulfill international consensus clinical and/or laboratory criteria as having “non-criteria obstetric APS” (13), since there is no data to justify such definition or even their treatment. Further studies are needed before we can suggest a new diagnostic criteria or an expansion with new inclusions for the current one.

## Pre-Eclampsia and Placental Insufficiency

Most of studies report a positive association between severe, early onset (prior to 34 weeks of gestation) pre-eclempsia (PreE), but the real frequency is unknown. Two published systematic reviews supported the association between aPL and PreE (6, 14). One of them (14) described a positive association between aPL and severe PreE in pregnant women without autoimmune disease, but there was no relationship with mild PreE. Both reported significant heterogeneity of study designs, definition of PreE, and study size.

Following these reviews, the APLA ObTF group updated the mentioned systematic analysis with more recent case-control studies that were published (8). All three antibodies were tested and only moderate-high titers were considered positive, although most of studies did not repeat testing to confirm results. Nevertheless, a positive association was found, especially with severe PreE.

Considering PI, publications are scarce, the definition is not well established, even in the obstetric arena, and inclusion criterion varies widely among studies. Most studies analyzing PI investigate aPL in patients with intrauterine growth restriction (IUGR), although other features of PI are outlined by the APS classification criteria (abnormal or non-reassuring fetal surveillance test(s), abnormal waveform analysis by Doppler flow suggestive of fetal hypoxemia and oligohydramnios).

The majority of cohort studies described a positive association between aPL and IUGR, but the small number of patients included and different definitions of IUGR and positive aPL should be underscored. Also, in fetuses with IUGR, it is difficult to exclude all confounding factors, including congenital infections, aneuploidy, or PreE, besides constitutionally small fetuses.

The association between aPL with PreE, especially severe PreE, and PI seems to be accurate, as both events can occur even with treated patients with established APS (4). The real frequency of aPL in these events, nonetheless, remains unknown, considering all issues previously stated. The most critical flaws of the studies are related to methodologies applied, and definition of positivity, heterogeneous definitions of PreE and PI, small sample size, and lack of repeat testing.

The ObTF proposed that a multicenter study to investigate aPL prevalence in patients PreE should be performed for a better understanding of the association. The design of a similar study considering PI, however, was considered difficult (8).

## Infertility

Infertility is not a criterion described in the international consensus (1), but investigation and treatment of aPL in infertile women is common in clinical practice. As aPL can affect throphoblast invasion and uterine decidualization, it was hypothesized that those antibodies could act before pregnancy was detected. However, this theory has never been confirmed.

A critical systematic review of the literature was conducted to analyze published studies regarding aPL positivity in infertile women, the association of aPL and *in vitro* fertilization (IVF) outcome, and the effects of medical treatments on IVF outcome of aPL positive women (8).

Thirteen of 29 studies (44%) assessing aPL prevalence in infertile women identified in that review reported a higher frequency compared to control populations. However, most of them investigated non-criteria aPL tests, which do not have current clinical significance, and only one used aPL cut-off as recommended. The presence of aPL did not influence the outcome of IVF in the majority of the studies, similar to a previous published meta-analysis result (15). Finally, treatment of aPL positive patients submitted to IVF was not beneficial.

Considering these results, the ObTF concluded that there is no evidence to support investigation of aPL in patients with infertility or treatment of aPL positive patients to improve results of IVF, except for research purposes. These conclusions are in consonance with recommendations of the American Society for Reproductive Medicine.

## Conclusion

Antiphospholipid antibodies are identified in a significant number of women with adverse obstetric events, such as recurrent miscarriages, fetal loss, pre-eclampsia, and PI. High quality scientific data to support these associations, however, are lacking, and future studies should address at least part of these uncertainties. The main limitation reported is the definition of positive aPL, with only few studies addressing this issue as recommended by the international classification criteria (1). Standardization of laboratory criteria and multicenter studies may help improve quality of following studies.

Investigation and treatment of aPL in a context different to that recommended by international APS criteria, such as two consecutive miscarriages, with low aPL titers or presenting infertility, should be reserved for research purposes, as current knowledge is insufficient to justify this conduct in daily practice or even inclusion in the current criteria.

## Conflict of Interest Statement

The authors declare that the research was conducted in the absence of any commercial or financial relationships that could be construed as a potential conflict of interest.
